# Breast Metastasis From Rectal Signet-Ring Cell Carcinoma: A Case Report and Review of Literature

**DOI:** 10.3389/fonc.2022.873354

**Published:** 2022-04-04

**Authors:** Yuran Dai, Yudi Jin, Ailin Lan, Nan Ding, Linshan Jiang, Shengchun Liu

**Affiliations:** ^1^ Department of Endocrine and Breast Surgery, The First Affiliated Hospital of Chongqing Medical University, Chongqing, China; ^2^ Department of Pathology, Chongqing University Cancer Hospital, Chongqing, China

**Keywords:** breast metastasis, rectal cancer, signet-ring cell carcinoma, breast cancer, case report

## Abstract

**Background:**

Metastatic rectal cancer (mRC) of the breast is an extremely rare clinical situation. There are few reported cases in domestic or foreign literature. The clinicopathologic characteristics along with the diagnostic and therapeutic strategies of such cases remain relatively unclear. Here, we would like to provide our comprehensive insights into this rare entity.

**Methods:**

We present a case that till now is the first reported breast metastasis from rectal cancer pathologically diagnosed as a signet-ring cell carcinoma, and we review the current literature on this rare event. The detailed clinical data, histopathology, management, and follow-up aspects were gathered for analysis.

**Results:**

A total of 15 cases were collected including the current case. Breast metastases from rectal cancer present at an average age of 47.7 years (range, 28 to 69 years) and appear with an average interval of 28.4 months (range, 5 months to 18 years) following primary tumor diagnoses. Of the 15 cases, 8 and 5 are pathologically diagnosed as adenocarcinomas and mucinous adenocarcinomas, respectively. Most cases (11/15) are accompanied by extramammary metastases. About half of the breast metastases (7/15) were to the left. In all cases, the main complaints were palpable mass. The average maximum diameter of the metastatic mass is 2.7 cm (range, 1–11 cm). The majority (8/12) of cases with accessible therapy information exclude the option of local surgery.

**Conclusion:**

Previous cancer history and accurate immunohistochemistry data play critical roles to distinguish mammary metastasis from a primary neoplasm of the breast. Mastectomy and molecular-targeted drugs should be considered with priority if systemic condition supports them.

## Introduction

The breast is rarely the site of metastatic disease. Of mammary malignancies, extramammary metastases account for only 0.43% (1). Among them, the primary lesions are derived mainly from the contralateral breast, and metastatic rectal cancer (mRC) forms a very small subset (2). Colorectal signet-ring cell carcinoma (SRCC) is also a rare entity, accounting for nearly 1% of all colorectal carcinomas (3). To our knowledge, this is the first case of breast metastasis arising from rectal cancer pathologically diagnosed as SRCC.

## Methods

The entire process of the diagnosis, management, and prognosis of this new case of mRC to the breast was reported to illustrate our thoughts about this rare disease.

In addition, we searched PubMed and Web of Science to identify all articles published in the English language, pertaining to breast metastases from rectal carcinoma. Only 15 cases including the present case have been reported to date (**Table 1**). Here we report a new case and review the cases (4–17) based on the current literature.

**Table 1:** Summary of cases; breast metastases from rectal cancer.

	Author/year	Sex/age	Primary rectal cancer		Breast metastasis					
			Pathology	Sites of organ metastasis	Laterality	Complaint	Size (cm)	Metastasis time	Management	Prognosis
1	Lal, R. L./1999 (4)	F/69	Moderately differentiated mucinous adenocarcinoma	Skin, liver, lung, brain	Left	Mass	NS	1 year	Surgery + chemotherapy	Deceased 4 months
2	David, O./2002 (5)	F/42	Small cell undifferentiated carcinoma	Posterior cervical lymph node	Bilateral	Mass	NS	2 years	NS	NS
3	Mihai, R./2004 (6)	F/53	Poorly differentiated adenocarcinoma	Lung, skin	Left	Mass	1	5 years	Chemotherapy	Observed 4 months
4	Hisham, R. B./2006 (7)	F/32	Poorly differentiated mucinous adenocarcinoma	Spine, eye, and orbit	Left	Mass	NS	10 months	Radiotherapy	Deceased 2 months
5	Wakeham, N. R./2008 (8)	F/45	Adenocarcinoma	Liver, lung	Bilateral	Mass	2–2.2	2 years	NS	NS
6	Li, H. C./2009 (9)	F/54	Poorly differentiated adenocarcinoma	Brain	Right	Mass	3.7	5 months	Chemotherapy	Deceased 7 months
7	Shackelford, R. E./2011 (10)	F/44	Poorly differentiated adenocarcinoma	Brain, lung	Left	Mass	11 * 8	7 years	NS	NS
8	Wang, T./2011 (11)	M/38	Mucinous carcinoma	Liver	Right	Mass	6.2 * 6	7 years	Chemotherapy + surgery	Deceased 6 months
9	Makhdoomi, R./2013 (12)	F/28	Mucinous adenocarcinoma	None	Bilateral	Mass	3 * 2/2 * 1	9 months	Chemotherapy	Observed 2 months
10	Ahmad, S. S./2019 (13)	F/43	Poorly differentiated adenocarcinoma	None	Right	Mass	2.6	NS	Chemotherapy + targeted therapy	NS
11	Cheng, X./2020 (14)	M/57	Low-grade adenocarcinoma	None	Right	Mass	3.9 * 3	8 months	Chemotherapy	Deceased 2 months
12	Gur, E. O./2020 (15)	M/47	Mucinous adenocarcinoma	None	Bilateral	Mass	1.5 * 1.2/1.9 * 0.9	2 years	Surgery + chemotherapy	Observed 6 months
13	Hasegawa, H./2020 (16)	F/67	Adenocarcinoma	Lung	Left	Mass	1 * 1	1 year	Chemotherapy + targeted therapy	Observed 5 months
14	Ye, Y. Y./2020 (17)	F/49	Poorly differentiated adenocarcinoma	Lung, liver, bone	Left	Mass	2.5 * 2	18 years	Radiotherapy + targeted therapy	Deceased 3 months
15	Current case	F/45	Poorly differentiated signet-ring cell carcinoma	Lung	Left	Mass	1.6 * 1	3 years	Surgery + chemotherapy + targeted therapy	Observed 18 months

Metastasis time: interval between rectal cancer and breast metastasis. Prognosis: prognosis after the diagnosis of breast metastasis. NS, not stated.

## Case Report

**Figure 1:**
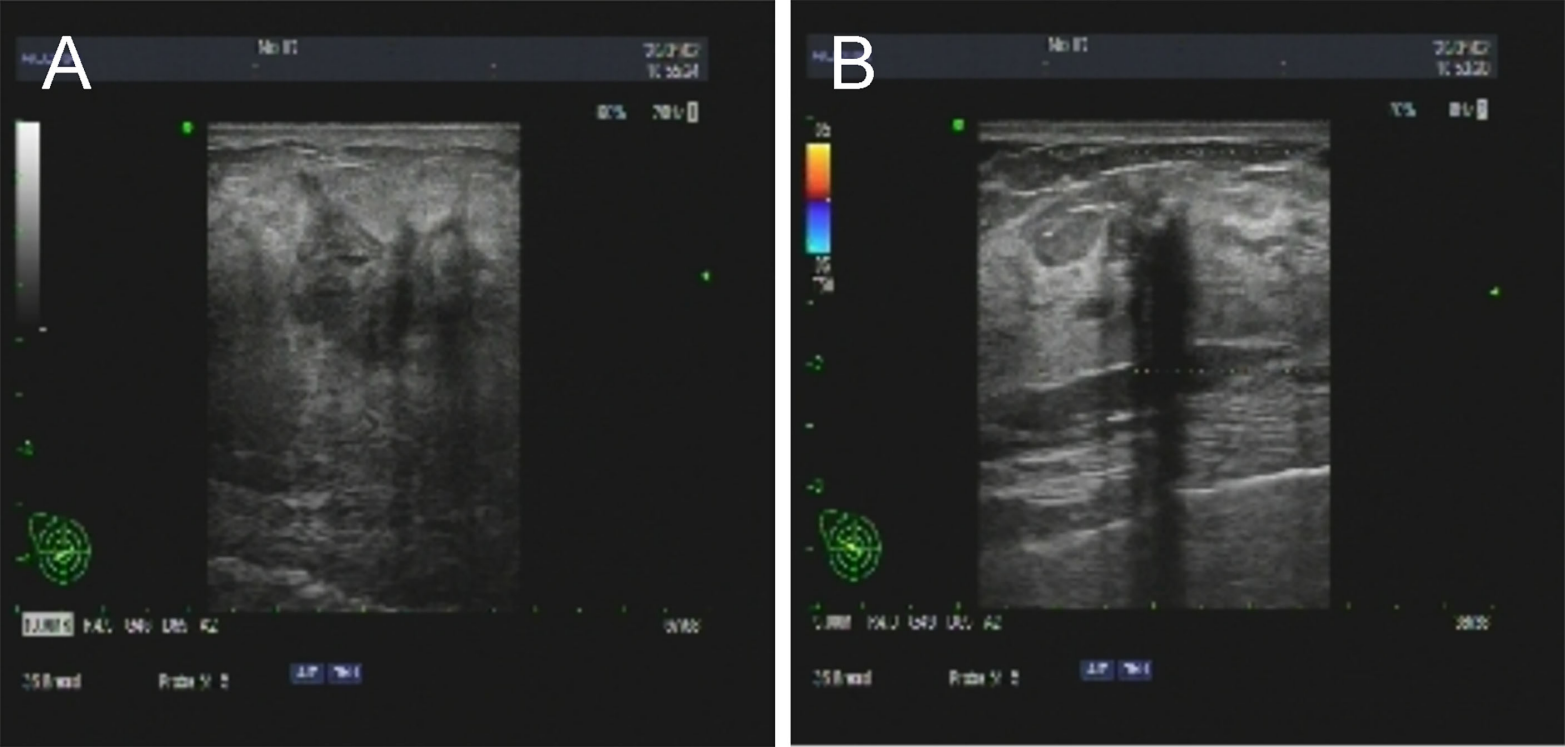
The ultrasound revealed 2 abnormal-echoic lesions in the left **(A)** and right **(B)** breast on which core needle biopsy was subsequently performed.

**Figure 2:**
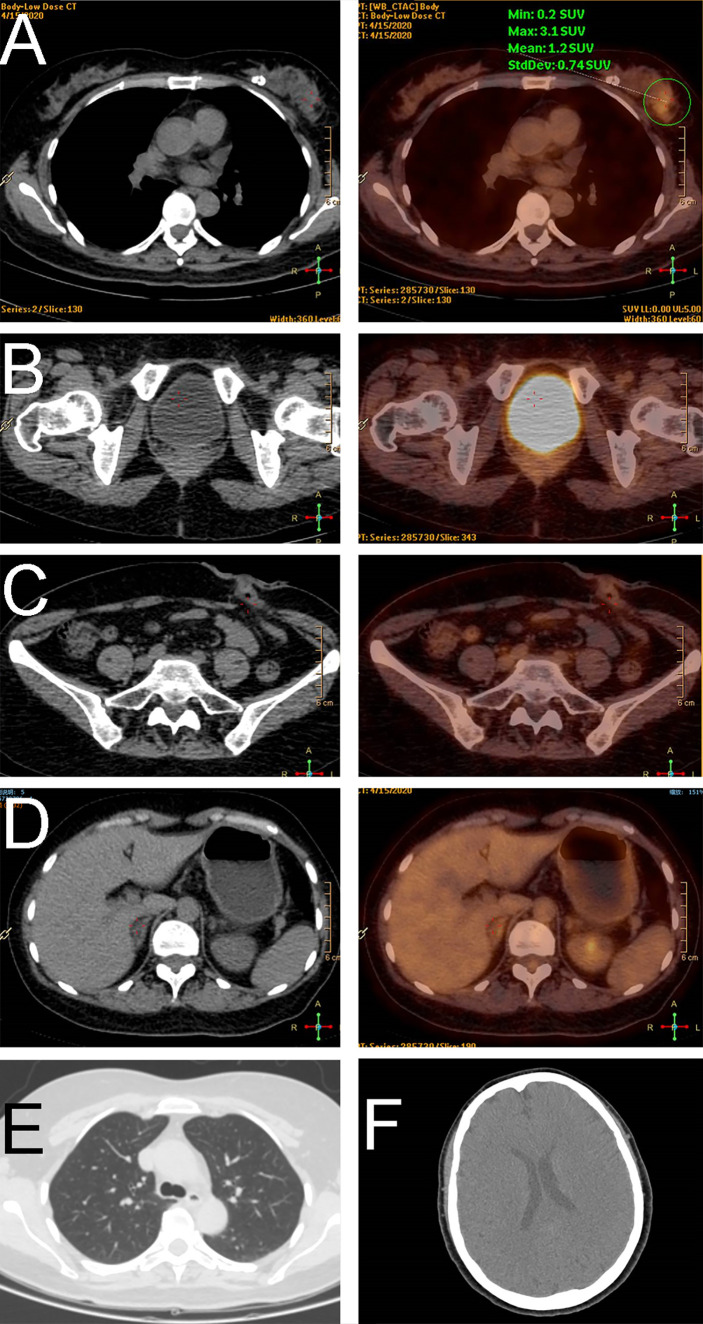
Positron emission tomography imaging demonstrated a tumor in the breast **(A)**, no relapse in the corresponding site of the rectum **(B)** or the colostomy site **(C)**, and no metastasis in the abdomen **(D)**, lung **(E)**, or head **(F)**.

**Figure 3:**
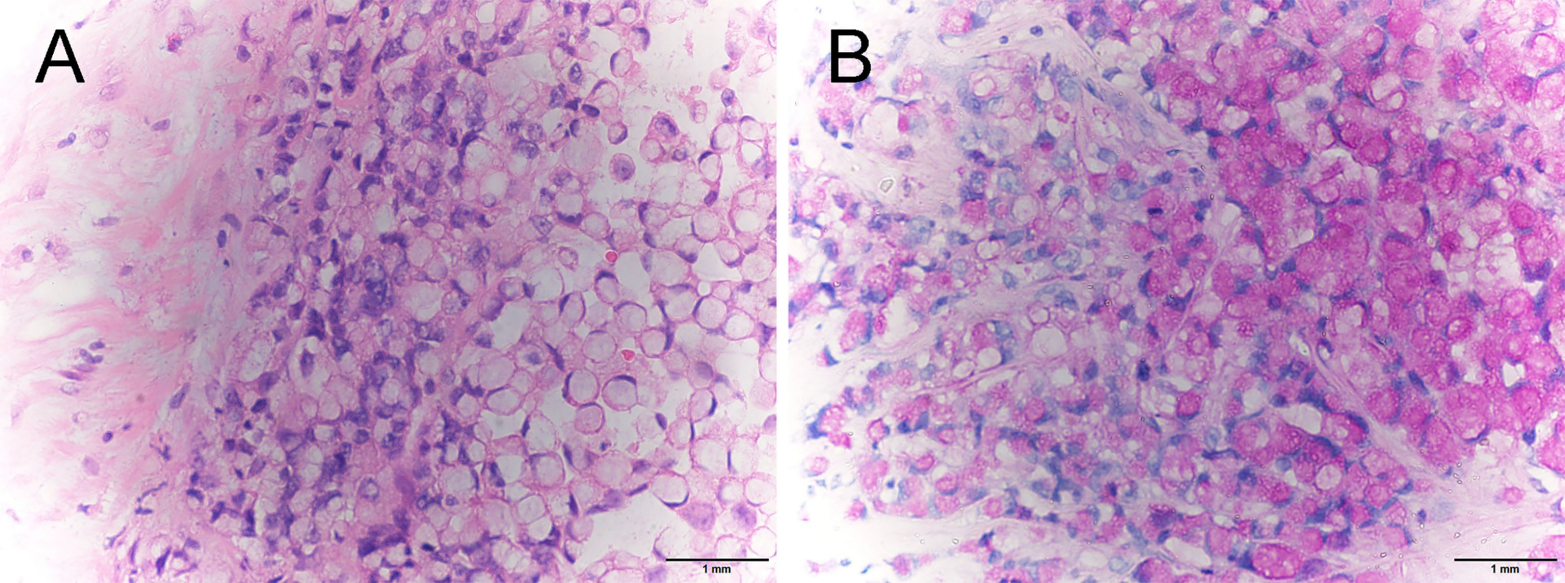
Pathological examination of breast specimen revealed diffuse infiltration of signet-ring cells (**A**, H&E stain, ×400) and intracytoplasmic lumens (**B**, PAS staining, ×400).

**Figure 4:**
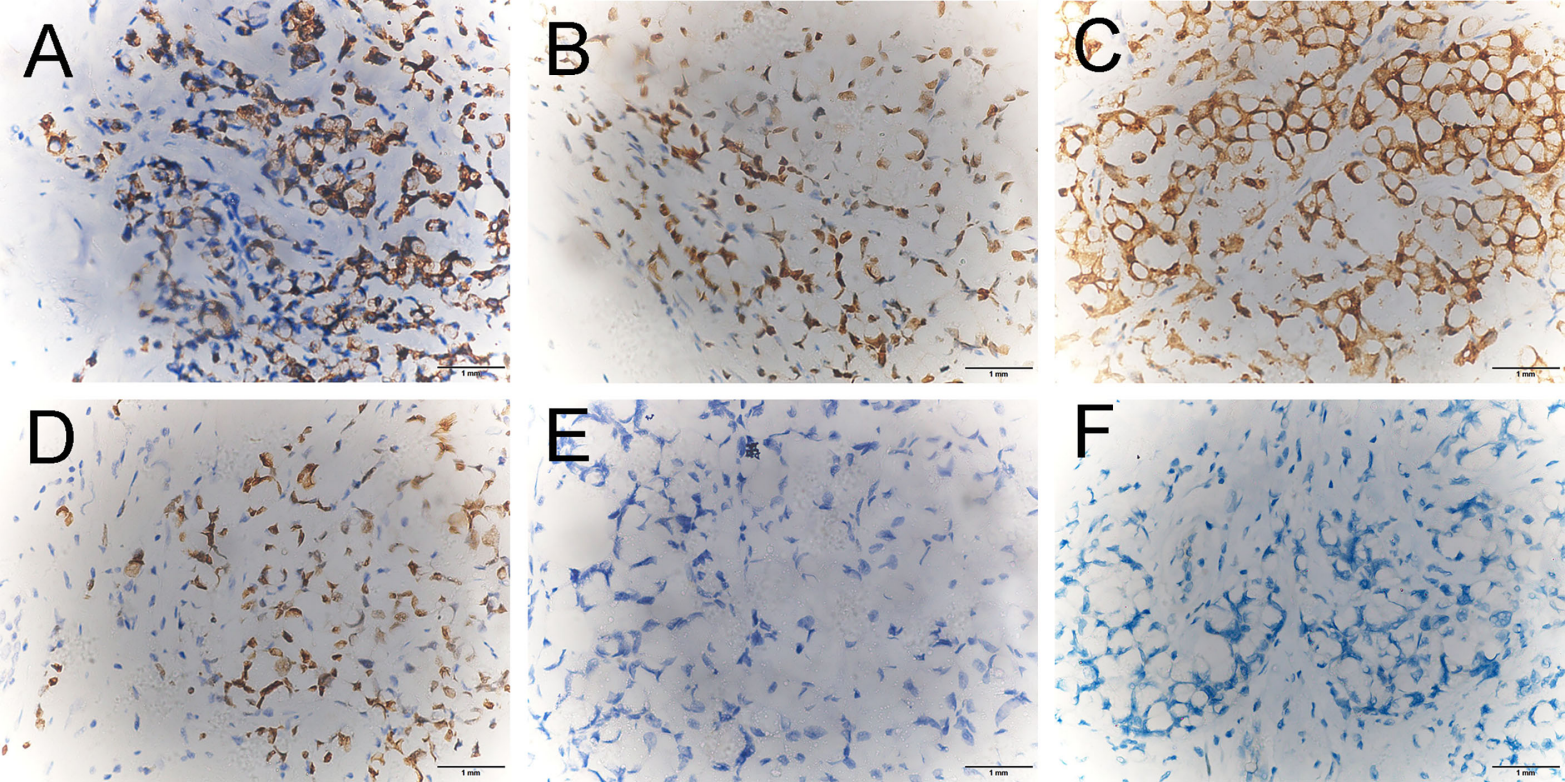
Immunohistochemical staining revealed that the breast tumor cells were positive for CK20 (**A** ×400), CDX-2 (**B** ×400), Villin (**C** ×400), and SATB-2 (**D** ×400) and were negative for estrogen receptor (ER) (**E** ×400) and progesterone receptor (PR) (**F** ×400).

A 45-year-old woman presented to us with a palpable mass in her left breast for 2 months. She had a history of rectal carcinoma for which she had undergone a Miles’ resection and subsequent systemic therapy 3 years previously. In her physical examination, a 6 * 6 cm firm lump with no pain or nipple discharge was palpated in the upper outer quadrant of the left breast. No axial lymph nodes were palpable. The ultrasound examination revealed 2 abnormal-echoic lesions in the left breast and 1 abnormal-echoic lesion in the right breast. Thereinto, the smaller one with multiple punctate echogenic foci in the left breast measuring 16 * 10 mm and the nodule of 10 * 9 mm in size in the right breast were both classified as BI-RADS 4A (**Figure 1**). The ultrasound-directed core needle biopsy was then performed on these two nodules. The cytological analysis demonstrated the nodule in the right breast as a benign lesion, while the nodule in the left breast was SRCC of intestinal origin, considering the patient’s history and immunohistochemistry (IHC) results. Routine blood investigations were unremarkable including carcinoembryonic antigen (CEA) and CA19‐9 levels. Positron emission tomography-CT (PET‐CT) indicated no evidence of malignancy or metastatic disease (**Figure 2**). Modified radical mastectomy was performed. The pathology of the resected specimen (**Figure 3**) reported an SRCC from the gastrointestinal tract in view of the IHC and the previous history. In IHC studies (**Figure 4**), estrogen receptor (ER)(−) and progesterone receptor (PR)(−) were reported. Cytokeratin (CK) expression with a pattern that is characteristic of colorectal tumors—CK20(+), CDX-2(+), Villin(+), and SATB-2(+)—were identified. Ki-67 index was 50%–60%. The postoperative recovery period was uneventful. Then the patient completed 6 cycles of the same chemotherapy containing calcium folinate, 5‐fluorouracil, and oxaliplatin (FOLFOX) as she received subsequent to the Miles’ resection. After 6 months of follow-up, a scan of the chest showed multiple solid lung nodules as evidence of lung metastasis. Therefore, the chemotherapy procedure was changed. Irinotecan was administered in combination with targeted therapies including angiogenesis inhibitor bevacizumab and epidermal growth factor receptor (EGFR) inhibitor cetuximab. Her systemic therapy is still ongoing, and there are no other distant metastases except those in the breast and lung after 18 months of follow-up.

## Results

Breast metastases originating from rectal cancer present at an average age of 47.7 years (range, 28 to 69 years), which is younger than the average of a primary colorectal cancer diagnosis at 72 years (18). On average, breast metastasis is reported to appear 28.4 months following rectal carcinoma diagnosis, while the interval varies between 5 months and 18 years.

Eight of fifteen cases are pathologically diagnosed as adenocarcinomas. Five are mucinous adenocarcinoma, of which two cases show features of signet-ring cell differentiation. However, there was insufficient evidence for these two cases to confirm clear diagnoses, making our case the first accurate diagnosed SRCC.

Eleven of fifteen cases have extramammary metastases before or after breast metastasis. On account of the short follow-up time of the rest of the cases, the probability of metastases to another organ besides the breast needs to be assessed more, resulting in a naturally poor prognosis. Williams et al. (2) demonstrated that metastases to the breast usually indicate disseminated disease, with a median survival of 10 months from the time of diagnosis of the breast metastasis. Thus, a systemic examination is strongly recommended. It is commonly the lung, liver, and brain where extramammary metastases were found.

Interestingly, based on the review of 19 cases of colorectal carcinoma metastasizing to the breast, Schaekelford et al. (10) reported that breast metastasis favors the left side. In accordance with the previous result, we demonstrate that half of the cases were to the left breast. This laterality may suggest the possibility of the presence of lymphatic preponderance instead of hematologic routes while both of them are common pathways for extramammary neoplasms to spread *via* (19, 20). Left lymphatic routes remind us of left supraclavicular lymph nodes, namely, Virchow’s nodes, which take their supply from lymph vessels in the abdominopelvic cavity. Furthermore, the features of lymphatic metastases have been reported for breast metastases originating from gastric and ovarian carcinomas (19). Taken together, the lymphatic pathway cannot be ruled out as an underlying mechanism of breast metastasis from rectal cancer. However, we could not find any relation between this preference and other clinicopathologic features. More study and observation about this phenomenon is necessary.

The clinical manifestations of all rectal cancer breast metastases reported in the literature are palpable mass consistently. Compared with that during primary breast cancer, the size of the metastatic mass (average 2.7 cm; range, 1–11 cm) is larger when initially diagnosed. The absence of carcinogenesis from carcinoma *in situ* to an invasive one is considered a vital reason. On the other hand, the exclusion of breast ultrasound from routine follow-up examinations of rectal cancer is also a contributing factor.

It is imperative to distinguish mRC to the breast from primary mammary malignancies, as the management is completely different. Fortunately, the combination of the previous history and the IHC results in the vast majority of cases are always able to give clues. As mentioned in the previous review (10), colorectal carcinoma is positive for CK20 and CDX2 and negative for breast markers ER and PR in greater than 90% of cases. In addition, subsequent studies (21–24) suggest that Villin and SATB2 can also be used as important complementary tools for the differential diagnosis of carcinoma of unknown primary origin. Here in our case, being positive for CK20, CDX-2, Villin, and SATB-2 strongly supports the rectal origin, as well as being negative for ER and PR.

In terms of management, eight of twelve cases with accessible therapy information chose palliative chemotherapy and/or radiotherapy without local surgery. Except for our case, there are only three cases in whom excisions were performed, where two cases were misdiagnosed as having primary breast cancer to undergo surgeries. This relatively conservative inclination is legitimate because of the exceedingly poor prognosis.

Nonetheless, due to the special pathological type of SRCC in the current case, we carefully thought over the individual strategy. Studies have shown that SRCC of the colorectum responds poorly to current cytotoxic treatments, and surgical management has a key role in the treatment of localized tumors (3). Metastatectomies have been increasingly performed for colorectal liver and lung metastases, as these have clearly improved the survival of patients whose primary disease has been controlled (9). Taking into consideration the histopathology and her good general condition at the time, modified radical mastectomy was finally performed followed by adjuvant chemotherapy of FOLFOX.

With the introduction of molecular-targeted drugs such as anti-EGFR monoclonal antibody therapies and angiogenesis inhibitors, treatment options for patients with metastatic colorectal cancer have changed considerably. As reported (25), with the assistance of targeted therapies, the median overall survival of metastatic colorectal cancer now reaches approximately 30 months instead of less than 1 year previously. In the current case, bevacizumab and cetuximab provide an additional 1 year of survival after lung metastasis was detected.

## Conclusion

mRC to the breast is an extremely rare clinical entity. By reviewing all reported cases, breast metastases from rectal carcinoma commonly 1) present as a palpable mass, 2) favor the left breast, 3) are accompanied by extramammary metastases, and 4) have a poor prognosis. A comprehensive reference to previous history and IHC data is crucially significant to establish a correct diagnosis. In patients with metastatic disease limited to the breast, or with minimal disease burden elsewhere, surgery intervention of breast metastases may be considered after a thorough systemic assessment, especially under the condition of a metastatic SRCC. Furthermore, molecular-targeted drugs should be administered aggressively as a promising therapeutic possibility.

## Author Contributions

YD and SL conceived the idea for the article. YD managed the case and drafted the manuscript. SL approved the final version of the manuscript. All authors contributed to the article and approved the submitted version.

## Conflict of Interest

The authors declare that the research was conducted in the absence of any commercial or financial relationships that could be construed as a potential conflict of interest.

## Publisher’s Note

All claims expressed in this article are solely those of the authors and do not necessarily represent those of their affiliated organizations, or those of the publisher, the editors and the reviewers. Any product that may be evaluated in this article, or claim that may be made by its manufacturer, is not guaranteed or endorsed by the publisher.
